# A dual-branch perception and hybrid attention integrated framework for temporal remote estimation of wheat leaf biomass

**DOI:** 10.3389/fpls.2026.1905545

**Published:** 2026-07-29

**Authors:** Haixia Li, Qian Li

**Affiliations:** 1 Huanghe Science and Technology University, Zhengzhou, China; 2 School of Geography and Environment, Jiangxi Normal University, Nanchang, China

**Keywords:** hybrid attention, leaf biomass, precision agriculture, remote sensing, unmanned aerial vehicle

## Abstract

**Introduction:**

Wheat leaf biomass is a key indicator of crop growth, nitrogen status, and yield potential, and its accurate estimation is essential for precision agriculture. Unmanned aerial vehicle (UAV) remote sensing provides multi-stage phenological observations for non-destructive biomass monitoring. However, existing approaches often fail to capture the superimposed temporal patterns inherent to crop phenology, including short-term physiological fluctuations driven by management events and long-term seasonal growth trends, as well as the cumulative causal effects of early-stage conditions on final biomass accumulation.

**Methods:**

This study proposed a dual-branch perception and hybrid attention integrated framework (DBAFN) for temporal estimation of wheat leaf biomass from UAV multi-temporal observations across key growth stages.

**Results and discussion:**

Experimental results demonstrated that the DBAFN achieved the best performance, with the coefficient of determination (R²) of 0.87, root mean square error (RMSE) of 38.41 g/m², mean absolute error (MAE) of 27.77 g/m², and relative RMSE (RRMSE) of 17.29%. Overall, the proposed framework provided an effective solution for temporal biomass estimation and demonstrated strong generalization capability, as further validated by independent experiments across different ecological regions and wheat genotypes (R² = 0.816-0.820). Compared with conventional machine learning models, the DBAFN showed consistently higher accuracy and lower prediction error. Multi-source feature analysis indicated that the combination of reflectance, vegetation indices, and canopy height provides the most accurate estimation. Ablation experiments further confirmed the effectiveness of each module in improving model performance. The SHapley Additive exPlanations (SHAP) analysis revealed that the canopy height and key spectral features contribute most to biomass prediction, highlighting the importance of integrating structural and physiological information. This study demonstrates that integrating multi-scale temporal dynamics, hybrid attention mechanisms, and transformer-based dependency modeling significantly improves the reliability of UAV-based biomass estimation. It offers a practical, data-driven pathway for intelligent crop monitoring and precision nitrogen management.

## 1 Introduction

Wheat (Triticum aestivum L.) is one of the most vital cereal crops globally, underpinning food security for billions. Leaf biomass, a key agronomic trait, serves as a direct indicator of photosynthetic capacity, nitrogen status, and ultimately, yield potential (Li et al., 2016; Fang et al., 2018; Yang et al., 2024). Traditional destructive sampling methods, while accurate, are labor-intensive, time-consuming, and spatially limited, rendering them unsuitable for large-scale, dynamic phenotyping (Safdar et al., 2023; Zhang et al., 2024). The advent of unmanned aerial vehicle (UAV) remote sensing has revolutionized high-throughput field phenotyping by enabling the rapid, non-destructive, and repeatable acquisition of high-resolution crop canopy data across critical growth stages (Sankaran et al., 2015; Yang et al., 2017). This technology provides a rich data stream, typically in the form of time-series imagery (e.g., RGB and multispectral), from which leaf biomass must be accurately estimated through robust analytical models.

However, transforming these multi-temporal UAV observations into reliable biomass estimates faces three intertwined methodological challenges that existing approaches struggle to address comprehensively. First, the multi-scale temporal dynamics inherent to crop growth are difficult to capture. In this study, we explicitly define “multi-scale variations” as the superimposed temporal patterns occurring at different phenological resolutions: (i) short-term physiological fluctuations driven by immediate environmental stimuli, and (ii) long-term phenological trends. Wheat biomass accumulation is not linear, but rather a combination of these two scales. It manifests as short-term fluctuations (e.g., rapid growth following irrigation or fertilizer application) superimposed upon long-term phenological trends (e.g., the sigmoidal pattern from tillering to grain filling) (Sakamoto et al., 2011; Dusfour-Castan et al., 2026; Ishaq et al., 2025). Conventional machine learning models, such as Random Forest (RF) or Support Vector Regression (SVR), often treat time-series data as static, handcrafted feature vectors (e.g., spectral indices per date), thereby discarding the crucial sequential order and temporal correlations (Feng et al., 2020; Mahmoud et al., 2025; Zhang et al., 2023). While simple one-dimensional Convolutional Neural Networks (1D-CNNs) can process sequences, their fixed receptive fields limit their ability to jointly model these disparate temporal scales effectively (Seydi et al., 2022; Cheng et al., 2024).

Second, there is a persistent challenge in adaptive feature selection and the suppression of redundant information. Research has heavily relied on predefined vegetation indices (VIs), such as the Normalized Difference Vegetation Index (NDVI). However, these indices often saturate in later growth stages under high biomass conditions and lack the adaptability to dynamically prioritize the most informative signals across different phenological phases (Nguy-Robertson et al., 2012; Cheng et al., 2024; Dronova and Taddeo, 2022). In a deep learning context, treating all feature channels equally can allow noise or less relevant temporal patterns to obscure critical signals, hindering model robustness and interpretability.

Third, modeling the long-range dependencies within a growth season is inherently difficult. In this study, we define “long-range dependencies” as the cumulative and causal effects of early-season physiological conditions (e.g., tillering density, early stress events) on late-season biomass accumulation. The final leaf biomass is a culmination of physiological processes and environmental interactions across the entire season (Dronova and Taddeo, 2022). For instance, early-season tillering density or a stress event during stem elongation can have prolonged effects on later biomass (Lobell et al., 2013; Zhao et al., 2024). Standard convolutional operations, with their localized focus, are inherently limited in establishing such distant temporal causal relationships. While Recurrent Neural Networks (RNNs) or Long Short-Term Memory (LSTM) networks are designed for sequences, they often suffer from gradient issues and can be less efficient at capturing very long-range dependencies compared to more recent architectures (Song and Wang, 2023; Yakut, 2025).

Recently, deep learning, particularly architectures combining convolutional neural networks (CNNs) and attention mechanisms, has shown significant promise in remote sensing and agricultural applications. For instance, to address spatiotemporal prediction tasks, studies have effectively combined spatial feature extraction with temporal sequence modeling (Liu et al., 2022; Zhang et al., 2025a). Similarly, lightweight CNN-LSTM architectures have been deployed for real-time yield forecasting on mobile devices (Gandotra et al., 2026), and deep learning models have been successfully applied to retrieve soil moisture and analyze its spatiotemporal variations (Zhang et al., 2025b). These studies demonstrate the growing capability of deep learning in handling spatiotemporal agricultural and environmental data.

However, despite these advances, most existing approaches still exhibit critical limitations when applied to crop growth dynamics. Many studies focus primarily on spatial feature extraction from single-date images or apply generic sequence models without explicitly tailoring them to the unique, multi-scale, phenology-aware nature of crop development. Specifically, the superposition of short-term environmental fluctuations (e.g., responses to irrigation or stress) and long-term seasonal trends, as well as the cumulative causal effects of early-season conditions on late-season biomass, remain insufficiently modeled. Furthermore, while attention mechanisms have been used to weigh feature importance, their integration for adaptive, stage-aware feature selection in crop phenology is still under-explored. Consequently, a dedicated framework that systematically integrates multi-scale temporal perception, adaptive feature refinement, and long-range dependency modeling for biomass estimation remains underdeveloped.

To bridge this gap, this study proposes a novel deep-learning framework specifically designed for the temporal remote estimation of wheat leaf biomass from UAV time-series data. The primary objectives and innovations are threefold: (1) To explicitly model multi-scale temporal dynamics, we introduce a Dual-Branch Temporal Perception Module. This module employs parallel convolutional pathways with different receptive fields to concurrently capture fine-scale fluctuations and broad phenological trends. (2) To achieve adaptive feature optimization, we design a Hybrid Attention Mechanism. This mechanism groups features by semantic similarity and employs a combination of average and max pooling to adaptively accentuate informative channels related to key growth events while suppressing noise, mimicking an agronomist’s focus on critical phases. (3) To capture complex long-range dependencies across the growth season, we incorporate a Transformer Encoder with Cross-Layer Feature Fusion. This allows the model to directly compute relationships between any two time points and integrates multi-level temporal representations for a more comprehensive understanding.

The remainder of this paper is organized as follows: Section 2 details the study area, UAV data collection, ground truth measurements, and the architecture of the proposed framework. Section 3 presents the experimental results, including model performance comparisons, ablation studies, independent validation, and visualizations of the model’s internal attention. Section 4 discusses the agronomic implications of the findings, the advantages of the integrated framework, and potential limitations. Finally, Section 5 concludes the paper and suggests directions for future work.

## 2 Materials and methods

### 2.1 Experimental design

**Figure 1:**
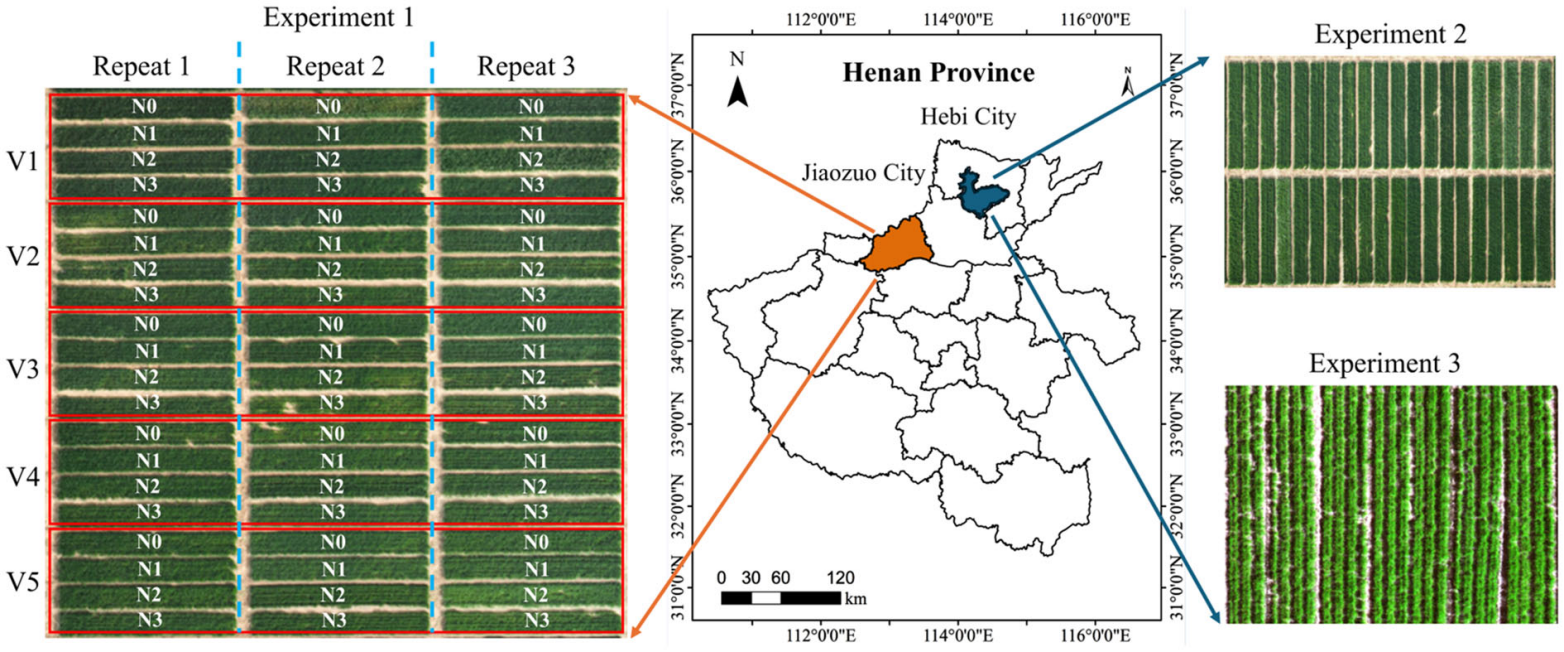
Experimental design and field layout.

The field experiment was conducted at an agricultural experimental station located in Jiaozuo, Henan Province, China (34°57′2″N, 112°58′41″E). The study area is situated in a typical winter wheat production region, characterized by a temperate continental monsoon climate, which provides suitable conditions for wheat growth. The experimental field was divided into 60 plots of equal size (1.5 m × 10 m), arranged in a regular layout to facilitate UAV-based image acquisition and ensure spatial consistency, as shown in **Figure 1**. To prevent cross-contamination between different nitrogen treatments, a physical buffer distance of 0.3 to 1.0 m was maintained between adjacent plots. Given the dryland agricultural conditions of the study area, the risk of surface runoff and lateral nutrient leaching was inherently minimal. These buffer zones further ensured the independence of each treatment during field operations. Each treatment was replicated three times to reduce the influence of soil heterogeneity and micro-topographic variability on the experimental results. A total of five wheat genotypes were included in the experiment, combined with four nitrogen application levels (0, 100, 200, and 300 kg·ha^-1^). These treatments were designed to simulate different nitrogen supply conditions and their effects on wheat growth and biomass accumulation. All field management practices, including sowing, irrigation, and pest control, were conducted according to local high-yield cultivation standards. This ensured consistency in crop management across all plots and improved the reliability and comparability of the collected data.

To rigorously evaluate the robustness and transferability of the proposed model across different ecological and agronomic conditions, two additional independent experiments were established in Hebi City (another distinct ecological region within Henan Province). Experiment 2 comprised 20 diverse wheat genotypes, each planted in two replicates, resulting in a total of 40 plots. This experiment was designed to assess model performance under broad genotypic variability without nitrogen treatment interference, reflecting a natural growth condition. Experiment 3 consisted of 25 plots of a single wheat cultivar, arranged uniformly to evaluate model consistency under homogeneous genetic background and management practices. Both experiments were managed under standard local cultivation conditions without imposed nitrogen gradients, representing typical farmer field scenarios.

### 2.2 UAV data acquisition and preprocessing

#### 2.2.1 UAV platform and multispectral sensor

UAV-based remote sensing data were acquired using a RedEdge-P multispectral camera (Micasense Co., Ltd., Seattle, Washington, USA) mounted on a multirotor UAV platform. The RedEdge-P sensor is a scientific-grade multispectral camera specifically designed for agricultural and environmental remote sensing applications, featuring five discrete spectral bands with narrowband scientific-grade filters. The sensor captures images at a resolution of 1456 × 1088 pixels per multispectral band (1.6 MP) and includes a global shutter to eliminate motion-induced distortion during flight. The five spectral channels and their center wavelengths are as follows: blue (475 nm), green (560 nm), red (668 nm), red edge (717 nm), and near-infrared (NIR, 842 nm), each with specific bandwidths (full width at half maximum) of 32 nm, 27 nm, 14 nm, 12 nm, and 57 nm, respectively. The inclusion of a red-edge band (717 nm) is particularly advantageous for vegetation monitoring, as it enables accurate estimation of chlorophyll content, nitrogen status, and crop stress responses.

#### 2.2.2 Flight mission design

Flight missions were carefully planned to ensure sufficient spatial coverage and image overlap for subsequent photogrammetric processing. The UAV was operated at a constant altitude of 60 m above ground level, resulting in a ground sample distance of approximately 20 mm per pixel. Both forward overlap and side overlap were set to 80% to ensure robust image alignment and seamless orthomosaic generation. The camera was configured to capture images at one-second intervals, with the triggering mode set to ensure consistent overlap across the entire survey area. All flights were conducted under clear sky conditions between 10:00 AM and 2:00 PM local time to minimize shadow effects and ensure stable illumination conditions.

A total of four UAV data collection campaigns were conducted at four critical phenological stages (jointing, booting, heading, and filling) throughout the wheat growing season to capture temporal variations in canopy development and biomass accumulation. The acquisition dates were March 25, 2025 (jointing stage), April 15, 2025 (booting stage), April 23, 2025 (heading stage), and May 20, 2025 (filling stage) in Experiment 1. The UAV data for Experiment 2 and Experiment 3 in Hebi City were acquired within a time window of no more than three days before or after each corresponding acquisition date of Experiment 1. These time points correspond to critical phenological stages for wheat growth, enabling comprehensive monitoring of leaf biomass dynamics from early vegetative development through grain filling.

#### 2.2.3 Image preprocessing and orthomosaic generation

Raw UAV images were processed using Agisoft Metashape Professional software (version 2.1.0, Agisoft LLC, Russia), a photogrammetric platform widely employed for generating high-quality three-dimensional models and orthomosaics from aerial imagery. The preprocessing workflow followed a standardized procedure consisting of several sequential steps to ensure radiometric and geometric accuracy.

First, radiometric calibration was performed using a calibrated reflectance panel (50% reflectance) to convert raw digital numbers (DN) to surface reflectance values (denoted as R475, R560, R668, R717, and R842 in this study). Reflectance panel images were captured immediately before and after each flight mission to account for variations in illumination conditions. The panel images were processed within Metashape to establish the reflectance calibration function, which was then applied to all acquired images, ensuring consistent radiometric correction across the entire dataset.

Following radiometric calibration, the image alignment step was conducted to estimate camera positions and generate a sparse point cloud. The software automatically detected common tie points across overlapping images and optimized camera parameters using bundle adjustment algorithms. Subsequently, a dense point cloud was generated through multi-view stereo reconstruction, which provided detailed three-dimensional information of the crop canopy surface. A digital elevation model (DEM) was then constructed from the dense point cloud using interpolation techniques. Finally, orthomosaics were generated by orthorectifying the calibrated images using the DEM, resulting in georeferenced, radiometrically calibrated reflectance maps for each of the five spectral bands.

#### 2.2.4 Vegetation indices calculation

To enhance the spectral information for biomass estimation, a suite of vegetation indices was calculated from the reflectance orthomosaics. These indices were selected based on their established sensitivity to vegetation biophysical parameters, including chlorophyll content, canopy structure, and leaf area index. For each sampling date, the indices were computed pixel-wise using the five spectral bands. The following vegetation indices were derived: Normalized Difference Vegetation Index (NDVI), Normalized Difference Red Edge Index (NDRE), Green Normalized Difference Vegetation Index (GNDVI), Optimized Soil Adjusted Vegetation Index (OSAVI), Enhanced Vegetation Index (EVI), Soil Adjusted Vegetation Index (SAVI), Chlorophyll Index Red Edge (CIre), and Simple Ratio (SR) (Chen et al., 2026; Luo et al., 2025).

#### 2.2.5 Canopy height extraction

Canopy height was derived from the difference between the digital surface model (DSM) and the digital terrain model (DTM) using a pre- and post-emergence differencing approach (Luo et al., 2022; Li et al., 2025). Specifically, a DSM representing the surface elevation including the crop canopy was generated from the dense point cloud of each acquisition date. A baseline DTM representing the bare soil surface was generated from imagery acquired prior to wheat emergence (pre-sowing). The canopy height model (CHM) was then calculated by subtracting the DTM from the DSM for each pixel. This approach effectively removes topographic variations and yields a spatially explicit representation of canopy height at each growth stage. The resulting CHM was resampled to match the spatial resolution of the reflectance orthomosaics and used as an additional input feature for biomass estimation.

### 2.3 Collection of wheat leaf biomass

To obtain ground-truth leaf biomass data for model training and validation, destructive sampling was conducted synchronously with each UAV flight campaign across the 60 experimental plots of Experiment 1. The same destructive sampling protocol was applied to Experiment 2 (40 plots) and Experiment 3 (25 plots) in Hebi City to collect ground-truth data for independent validation. At each sampling time point, aboveground plant materials were collected from a representative area of 0.2 m × 0.2 m within each plot, avoiding border effects. The collected plant samples were immediately placed into labeled paper bags and rapidly transported to the laboratory to minimize biomass loss.

In the laboratory, the leaf component was separated from the remaining aboveground biomass. The leaf samples were placed in an oven and dried at 105 °C for 30 min to deactivate enzymatic activity, followed by continuous drying at 75 °C until a constant weight was achieved. The dry weight of the leaf material was recorded using an electronic balance with a precision of 0.01 g. The leaf biomass per unit area (g/m²) was then calculated by dividing the dry weight by the sampling area. This process was repeated at each growth stage to capture temporal variations in leaf biomass accumulation.

### 2.4 Dual-branch attention-based fusion network

**Figure 2:**
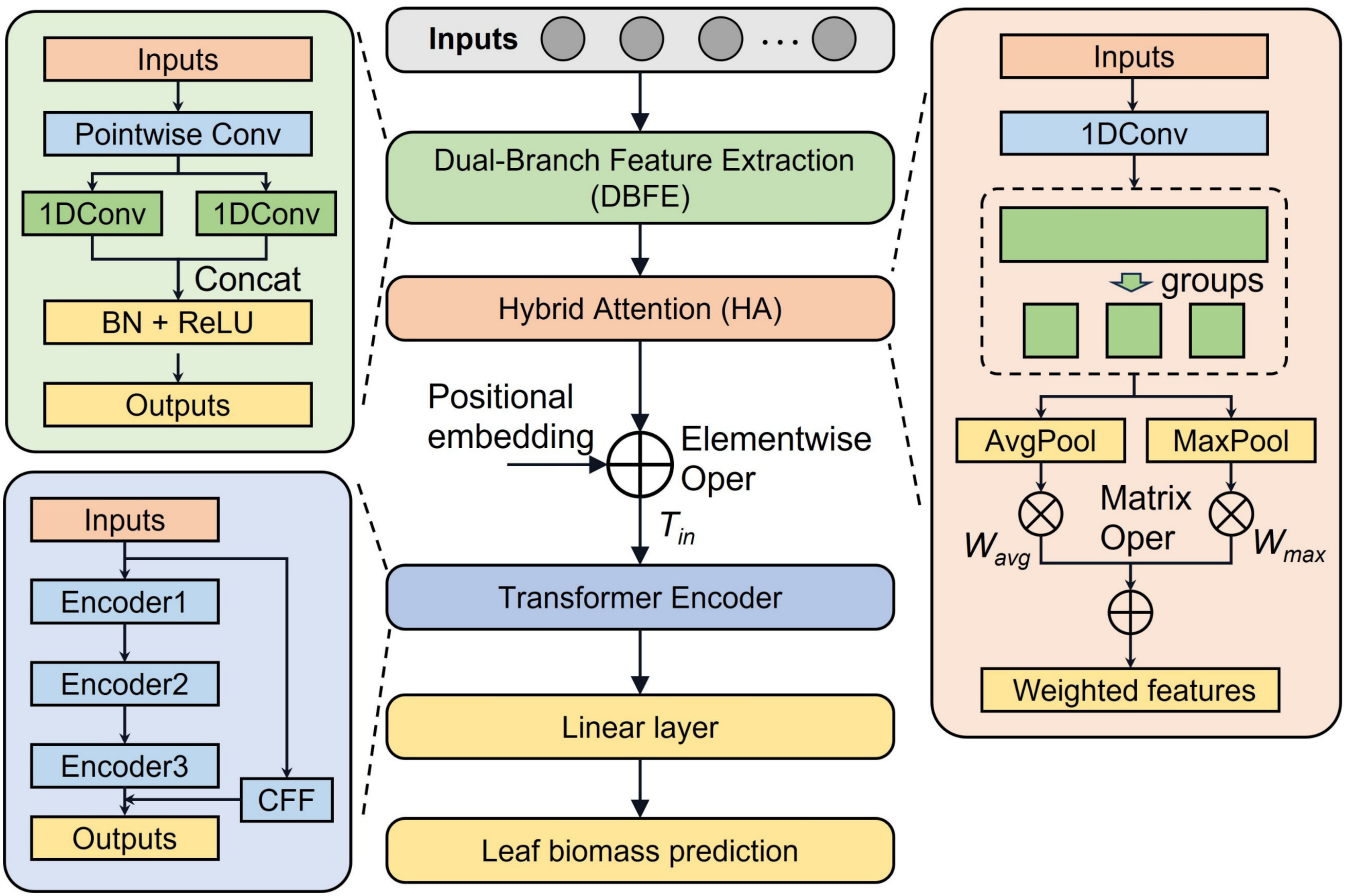
Architecture of the proposed dual-branch attention-based fusion network (DBAFN).

**Figure 2** illustrates the overall workflow of the proposed Dual-Branch Attention-Based Fusion Network (DBAFN), which is developed to predict wheat leaf biomass from UAV data. The model is designed to extract complementary patterns by processing the input through two parallel feature extraction branches. Each branch operates under a different receptive field scale, enabling the network to learn both fine-grained fluctuations and broader phenological trends related to biomass accumulation. The features produced by the dual branches are then merged and passed into a hybrid attention mechanism, which adaptively emphasizes informative patterns while suppressing redundant or weak signals. This attention process helps guide the model to focus on feature dimensions that are more biologically relevant to wheat growth dynamics.

#### 2.4.1 Dual-branch feature extraction

To capture multi-scale correlations among heterogeneous UAV-derived features, a one-dimensional dual-branch convolution module was developed. The module consists of four stages: pointwise channel projection, parallel temporal convolution, feature concatenation, and activation. Given the input vector sequence $\hat{X}$ consisting of multi-source UAV-derived variables, the input tensor of DBAFN is organized as follows (Equation 1):

(1) $$\hat{X}=R^{B\times F}$$

where B represents the batch size and F represents the number of input features. The first step applies a pointwise convolution to adjust the latent feature dimensionality (Equation 2):

(2) $$h_{0}={\text{Conv}}_{1\times 1}\left(\hat{X}\right)$$

The output *h*_0_ is processed by two parallel 1D convolution branches with different receptive fields. The first branch extracts short-term fluctuations using a small kernel size *k_s_*, while the second branch captures broader temporal trends with a larger kernel size *k_l_* (Equation 3):

(3) $$h_{s}=f\left({\text{Conv}}_{k_{s}}\left(h_{0}\right)\right),\;h_{l}=f\left({\text{Conv}}_{kl}\left(h_{0}\right)\right)$$

The outputs *h_c_* of both branches are concatenated along the channel dimension (Equation 4):

(4) $$h_{c}=\left[h_{s}∥h_{l}\right]$$

A batch normalization layer followed by a ReLU activation is then applied to stabilize training and enhance feature nonlinearity. Finally, this module structure allows the model to jointly leverage fine-scale vegetation changes and long-term growth patterns, which improves its ability to represent biomass-related temporal dynamics.

#### 2.4.2 Hybrid attention

The Hybrid Attention (HA) mechanism aims to identify informative channels by combining general trend information and critical peak variations, both of which are important for characterizing vegetation growth and predicting leaf biomass. Given the input feature map, the feature channels are first divided into different groups. This grouping operation encourages each subset of channels to focus on different semantic patterns, such as vegetation greening rate, peak greenness timing, or senescence dynamics, which are closely related to biomass accumulation.

Then, for each group, two types of temporal pooling are computed: average pooling and max pooling. Average pooling reflects the overall trend and can be interpreted as a representation of continuous but vague memory from repeated growth stages, while max pooling highlights sharp variations and can be viewed as memory of significant events such as rapid leaf expansion or stress response. Given the input *X*, the pooling operations are as follows (Equation 5):

(5) $$Z_{avg}=GAP\left(X\right),\;\;Z_{max}=GMP\left(X\right)$$

The two statistics are then combined and passed through a shared fully connected transformation (Equation 6):

(6) $$Z=\sigma \left(W\left(Z_{avg}+Z_{max}\right)\right)$$

Where *W* is a learnable weight matrix and σ denotes the sigmoid activation. Finally, the learned attention weights are broadcast and applied back to the grouped feature representation (Equation 7):

(7) $$O_{att}=Z⊙X$$

The HA module enhances the expressiveness of intermediate representations. This design is particularly beneficial for wheat leaf biomass prediction because biomass accumulation is influenced not only by gradual vegetation growth but also by peak physiological responses such as rapid leaf expansion or stress-induced decline. Therefore, combining memory-like average pooling with event-sensitive max pooling improves the network’s ability to capture biologically meaningful temporal cues, leading to more stable and accurate biomass estimation.

#### 2.4.3 Transformer encoder

The output of the aforementioned HA module is further processed by a transformer encoder block to model long-range dependencies and semantic interactions among features. The transformer encoder consists of three stacked encoding layers, each containing a multi-head self-attention (MHA) unit followed by a position-wise feed-forward network (FFN). For each layer, residual skip connections and layer normalization are applied to maintain stability during training and preserve original feature information. Formally, for the i-th encoder layer, the forward process is expressed as Equation 8:

(8) $$\begin{array}{c} T_{i}^{\prime }=\text{MHA}\left({\text{T}}_{i-1}\right)+T_{i-1}\\ T_{i}=\text{FFN}\left({\text{T}}_{i}^{\prime }\right)+T_{i}^{\prime } \end{array}$$

where *T_i_*_−1_ denotes the input to the layer and *T_i_* is its output. To strengthen information flow across the stacked encoders, a cross-layer feature fusion (CFF) mechanism was introduced. Instead of relying solely on the final encoder output, the fused representation integrates both shallow and deep encoded features. This adaptive fusion can be formulated as Equation 9:

(9) $$T_{fusion}=\sum_{i=1}^{3}\alpha_{i}T_{i},\;\sum_{i=1}^{3}\alpha_{i}=1$$

Where *α_i_* are learnable weights. This strategy helps retain fine-grained temporal cues captured by earlier layers while leveraging high-level semantic abstraction from deeper layers. As wheat leaf biomass is influenced by gradual seasonal progression as well as rapid crop-growth transitions, such hierarchical temporal encoding supports more robust and biologically meaningful prediction.

#### 2.4.4 Hyperparameters setting

The model input consisted of 14 UAV-derived features, including canopy height, spectral reflectance, and vegetation indices. For each sample, the input tensor was organized as *B*×14, where B represents the batch size. Before feature extraction, the input was reshaped into *B*×14 for one-dimensional convolution operations. The dual-branch feature extraction module employed a pointwise convolution followed by two parallel convolution branches with kernel sizes of 3 and 5, respectively. The projected feature dimension was set to 64. After concatenation, batch normalization and ReLU activation were applied. The hybrid attention module divided feature channels into four groups and combined average pooling and maximum pooling to generate adaptive channel weights. The Transformer encoder consisted of three layers with four attention heads. The embedding dimension and feed-forward dimension were set to 64 and 128, respectively. During training, the model was optimized using AdamW with an initial learning rate of 0.001 and weight decay of 1×10^−4^. Mean squared error was used as the loss function. The batch size was set to 32, and the maximum training epoch was 500. Early stopping was applied with a patience of 60 epochs. All experiments were conducted with a fixed random seed of 42 to ensure reproducibility.

### 2.5 Baseline model and performance evaluation

To evaluate the effectiveness of the proposed DBAFN model, three widely used machine learning methods were selected as baseline models, including random forest regression (RFR), support vector regression (SVR), and least absolute shrinkage and selection operator regression (LASSO). These models were used for comparison because they have been widely applied in agricultural remote sensing and biomass estimation tasks.

Model hyperparameters were optimized using RandomizedSearchCV. The search spaces of the main parameters for each baseline model are listed in **Table 1**. For RFR, the number of boosting rounds, maximum tree depth, minimum number of samples for each split, and minimum number of samples for each node were optimized. For SVR, the kernel type, penalty coefficient C, and gamma were tuned. For LASSO, the regularization parameter alpha was optimized. Randomized search was adopted to improve search efficiency and reduce the computational cost of parameter tuning.

**Table 1:** Parameters optimization of baseline models.

Model	Parameter	Search space
RFR	Number of boosting rounds	randint(100, 1500)
	Maximum tree depth	randint(3, 10)
	Minimum number of samples for each split	randint(3, 10)
	Minimum number of samples for each node	randint(3, 10)
SVR	Kernel	[rbf, linear, poly]
	C	[0.1, 1, 10, 100]
	gamma	[0.001, 0.01, 0.1, 1, 10]
LASSO	alpha	[0.001, 0.005, 0.01, 0.05, 0.1, 0.5, 1]

In terms of model construction in Experiment 1, all samples were divided into training and testing sets at a ratio of 7:3. The training set was used for model training and validation, while the testing set was used for independent evaluation. In addition, different input variable combinations were further tested to examine their effects on model performance. These combinations included single-source variables and multi-source combinations among reflectance (R), vegetation indices (VI), and canopy height (CH).

Model performance was finally evaluated using the coefficient of determination (R^2^), root mean square error (RMSE, g/m^2^), mean absolute error (MAE, g/m^2^), and relative root mean square error (RRMSE). R^2^ was used to measure the goodness of fit between predicted and observed values. RMSE and MAE were used to quantify prediction errors, while RRMSE was used to assess the relative prediction error. These metrics were jointly used for model comparison and accuracy evaluation. It is worth noting that the 7:3 random splitting strategy employed in this study is a widely adopted practice in agricultural remote sensing and biomass estimation research (Zhen-qi et al., 2023; Yue et al., 2019). To ensure the reproducibility and minimize the influence of random variation, a fixed random seed was used during the data partitioning process. Additionally, the robustness and generalization of the proposed model were further validated through independent datasets from different ecological regions (Experiments 2 and 3), as detailed in Section 3.4.

### 2.6 SHAP interpretability analysis

To interpret the decision-making process of the proposed DBAFN model and identify the key features driving the biomass estimation, we employed the SHapley Additive exPlanations (SHAP) framework (Lundberg and Lee, 2017). SHAP is a game-theoretic approach that assigns an importance value to each input feature for a given prediction, ensuring local accuracy and consistency.

The SHAP analysis was implemented using the Python shap library (version 0.42.0). Specifically, we utilized the DeepExplainer algorithm, which is optimized for deep neural network models to efficiently approximate Shapley values. The analysis was performed on the independent test dataset (30% of the total samples) to reflect the model’s predictive behavior on unseen data.

To evaluate global feature importance, we calculated the mean absolute SHAP value for each input variable across all test samples, with larger values indicating greater contributions to the model output. Furthermore, we generated SHAP summary plots and heatmaps to visualize the distribution of feature contributions and their sample-specific variations. This comprehensive SHAP framework provides quantitative and visual insights into how the DBAFN model leverages multi-source features for wheat leaf biomass estimation.

## 3 Results

### 3.1 Model performance overview

**Figure 3:**
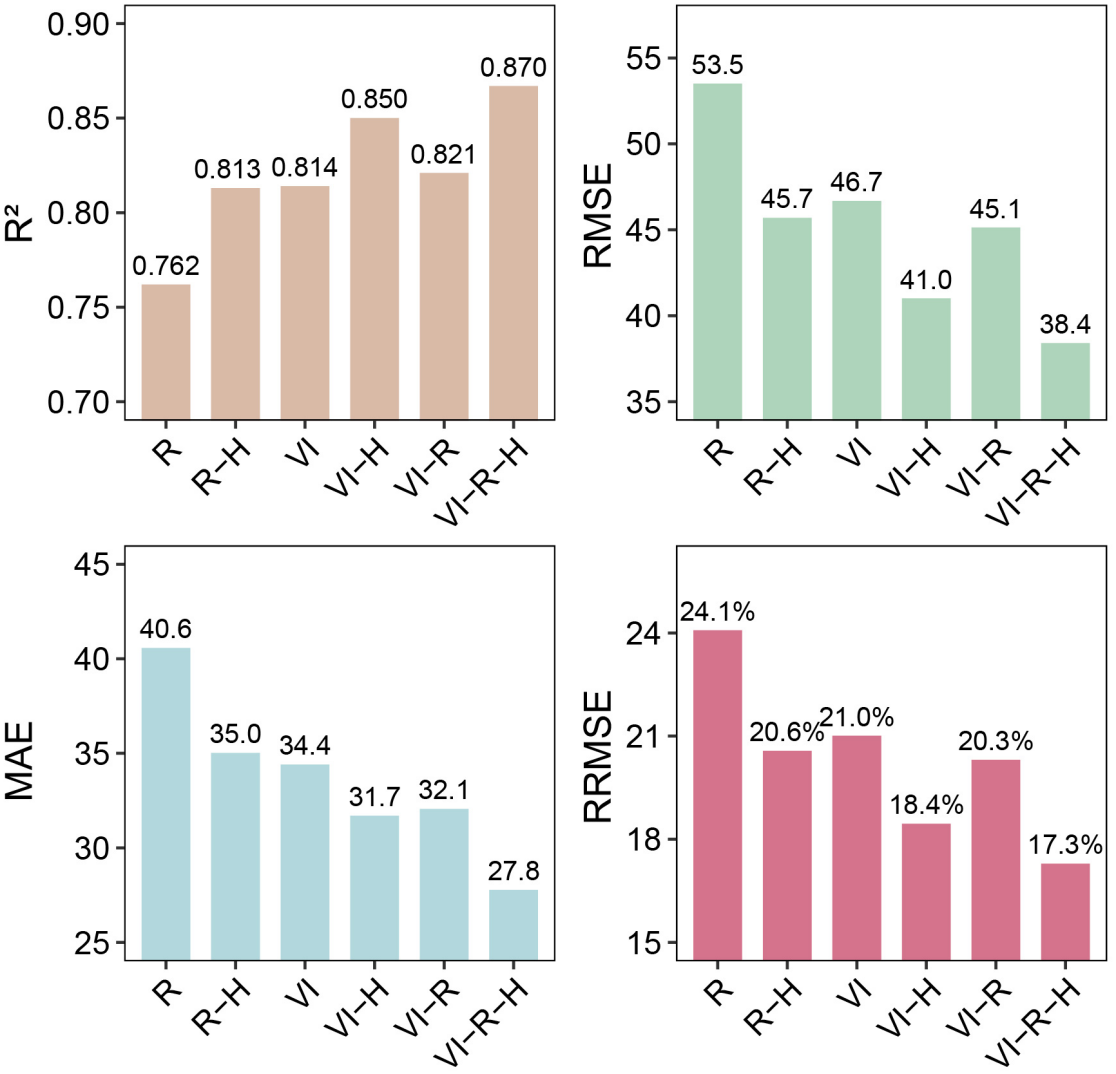
Prediction performance of different variable combinations. R denotes spectral reflectance; VI denotes vegetation indices; CH denotes canopy height. RMSE is the root mean square error; MAE is the mean absolute error; RRMSE is the relative root mean square error.

**Figure 3** shows the prediction performance of wheat leaf biomass under different variable combinations, including reflectance, vegetation indices, and canopy height. Clear differences can be observed across the evaluation metrics. When only reflectance is used, the model achieves the lowest accuracy, with an R^2^ of 0.762 and relatively high RMSE, MAE, and RRMSE values. This indicates that spectral information alone is not sufficient to fully describe wheat leaf biomass. The introduction of vegetation indices or canopy height leads to a clear improvement. The combination of reflectance and canopy height performs better than reflectance and vegetation indices, suggesting that the structural information provides more direct support for biomass estimation. A similar trend can be observed in MAE and RMSE, where the inclusion of height reduces prediction errors more effectively.

When vegetation indices and the canopy height are combined, the model achieves further improvement, with R^2^ increasing to 0.850 and RMSE decreasing to 41.0 g/m^2^. This result indicates that the spectral features and structural features are complementary and can jointly enhance the model performance. The best performance is obtained when all three types of variables are used together. The R^2^ reaches 0.870, while RMSE, MAE, and RRMSE decrease to 38.4%, 27.8%, and 17.3%, respectively. This demonstrates that integrating the reflectance, vegetation indices, and canopy height provides the most comprehensive representation of wheat growth status. Overall, these results confirm that multi-source feature fusion is essential for accurate estimation of wheat leaf biomass, and that structural information plays a key role in improving prediction performance.

**Figure 4:**
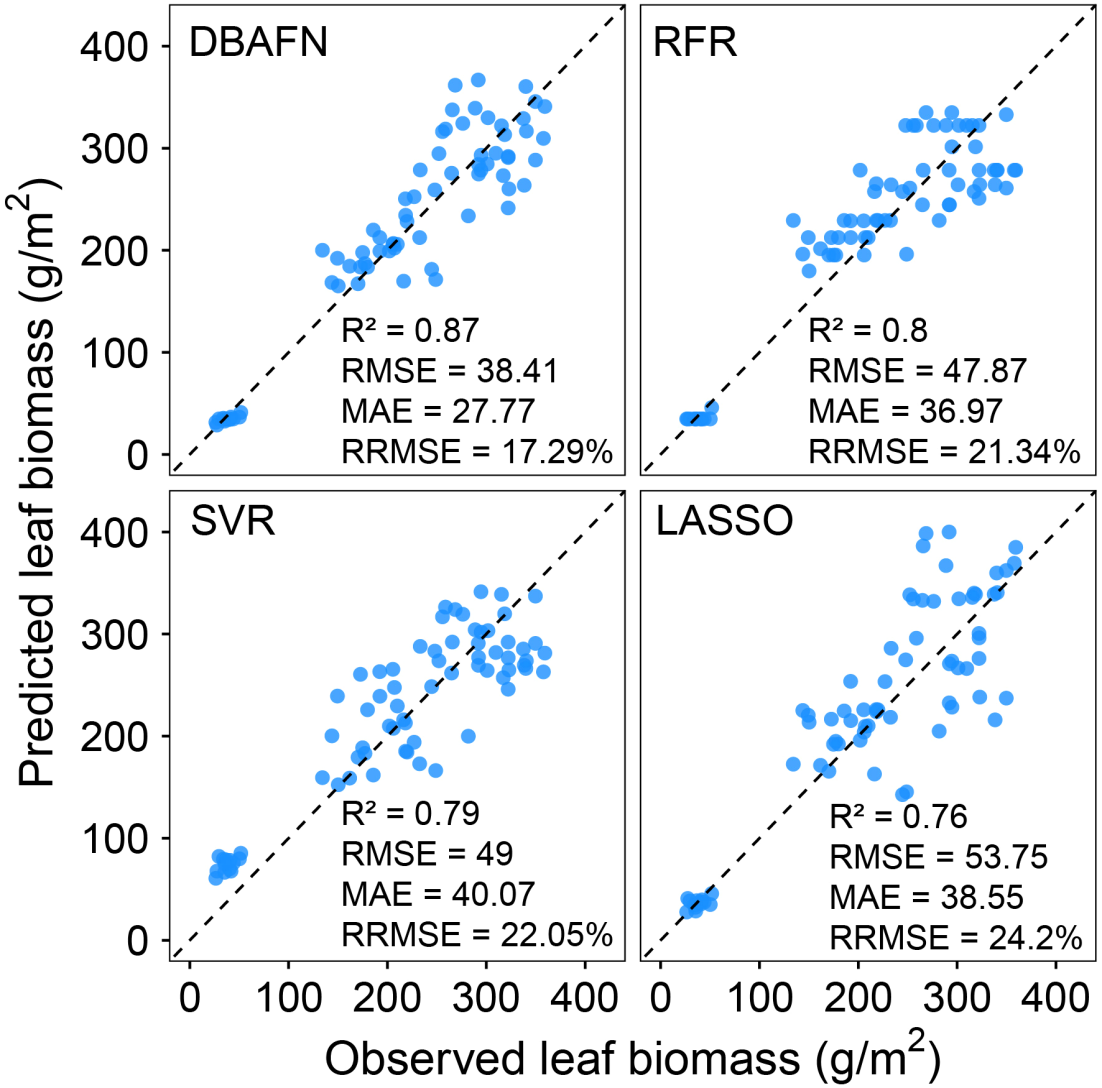
Prediction performance comparison of different models. DBAFN is the proposed Dual-Branch Attention-Based Fusion Network; RFR is Random Forest Regression; SVR is Support Vector Regression; LASSO is Least Absolute Shrinkage and Selection Operator regression. R² is the coefficient of determination; RMSE is the root mean square error (g/m²); MAE is the mean absolute error (g/m²); RRMSE is the relative root mean square error (%).

The comparison between DBAFN and the benchmark models for wheat leaf biomass estimation is shown in **Figure 4**. DBAFN shows the best agreement with observed values, with most points concentrated near the 1:1 line. More specifically, DBAFN achieves the highest accuracy, with an R^2^ of 0.87, RMSE of 38.41 g/m^2^, MAE of 27.77 g/m^2^, and RRMSE of 17.29%. In contrast, RFR, SVR, and LASSO obtain lower R^2^ values of 0.80, 0.79, and 0.76, respectively, and higher prediction errors. RFR performs better than SVR and LASSO, but still shows larger dispersion, especially at higher biomass levels. SVR further increases the prediction error, while LASSO exhibits the weakest performance with the largest deviation from the 1:1 line. Overall, DBAFN provides more accurate and stable predictions, indicating its stronger ability to capture the relationship between input features and wheat leaf biomass. It should be noted that, for brevity and clarity, the performance comparison between the proposed DBAFN and the benchmark models (RFR, SVR, LASSO) is presented only under the optimal multi-source feature set (R+VI+CH), as this configuration was shown to provide the best predictive capability in the feature ablation analysis. This approach allows for a direct and fair assessment of algorithmic effectiveness while maintaining a focused narrative.

### 3.2 Model interpretability with SHAP technology

**Figure 5:**
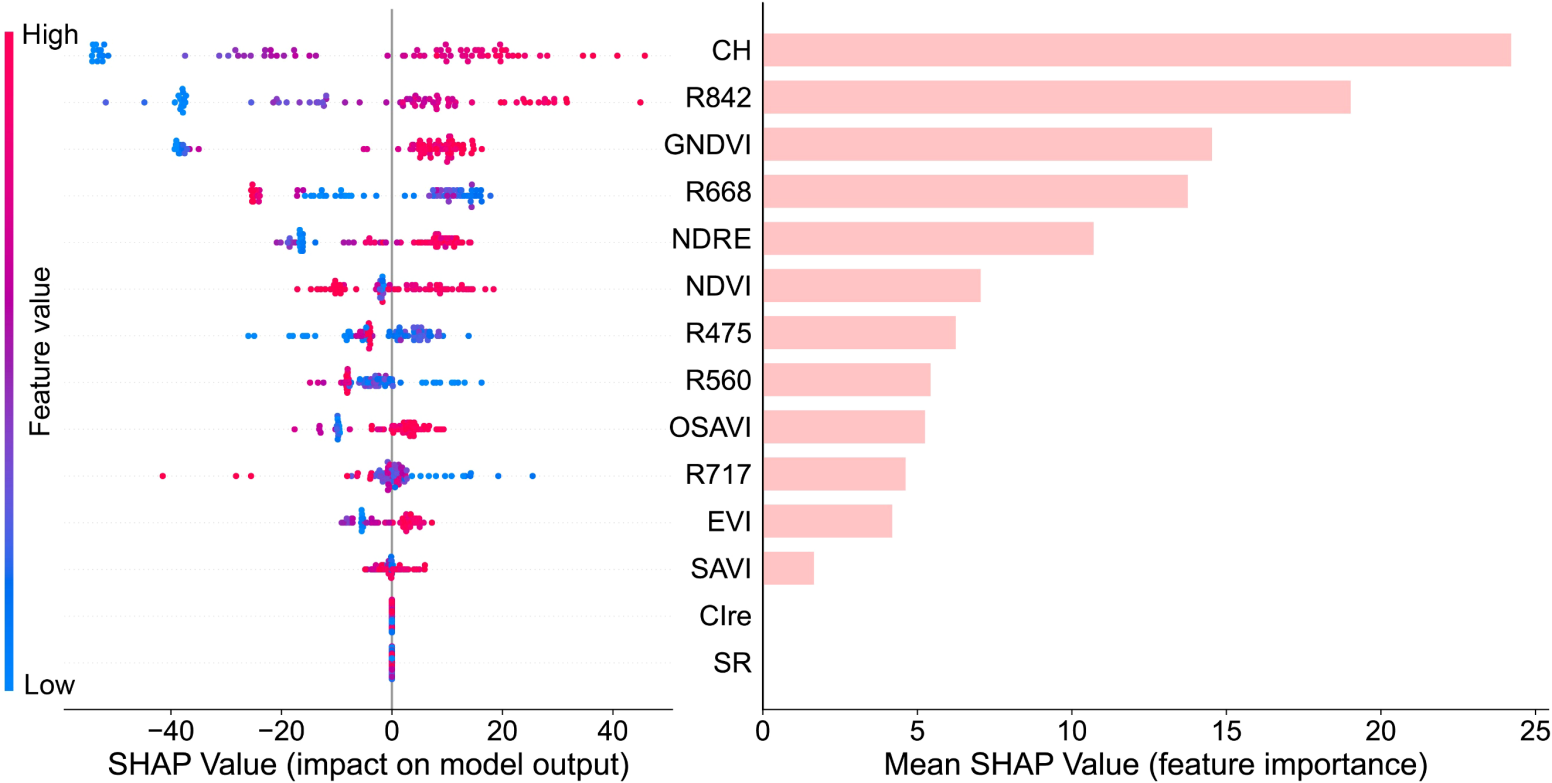
Feature importance analysis results for the DBAFN model using the SHAP technique. CH denotes canopy height; GNDVI is the Green Normalized Difference Vegetation Index; NDRE is the Normalized Difference Red Edge Index; NDVI is the Normalized Difference Vegetation Index; OSAVI is the Optimized Soil Adjusted Vegetation Index; EVI is the Enhanced Vegetation Index; SAVI is the Soil Adjusted Vegetation Index; CIre is the Chlorophyll Index Red Edge; SR is the Simple Ratio. R475, R560, R668, R717, and R842 represent the spectral reflectance at 475, 560, 668, 717, and 842 nm, respectively.

**Figure 5** shows the SHAP-based feature importance results of DBAFN model. Clear differences can be observed among the input variables. Canopy height has the largest mean SHAP value, indicating that it is the most important variable for leaf biomass estimation. Among the spectral variables, R842 shows the second highest contribution, followed by GNDVI, R668, and NDRE. NDVI, R475, R560, OSAVI, R717, and EVI also contribute to the model, but their effects are relatively weaker. In contrast, SAVI, CIre, and SR have very limited contributions. The SHAP summary plot further shows the direction of feature effects. High canopy height values generally produce positive SHAP values, indicating a positive contribution to biomass prediction. A similar pattern is also observed for R842 and GNDVI, where higher feature values tend to increase the predicted leaf biomass. For some variables, such as R668 and NDVI, both positive and negative effects are observed, suggesting more complex relationships with biomass. Generally, the SHAP results indicate that DBAFN mainly relies on canopy structural information and key spectral features for prediction. This result supports the importance of combining height, reflectance, and vegetation information in wheat leaf biomass estimation.

**Figure 6:**
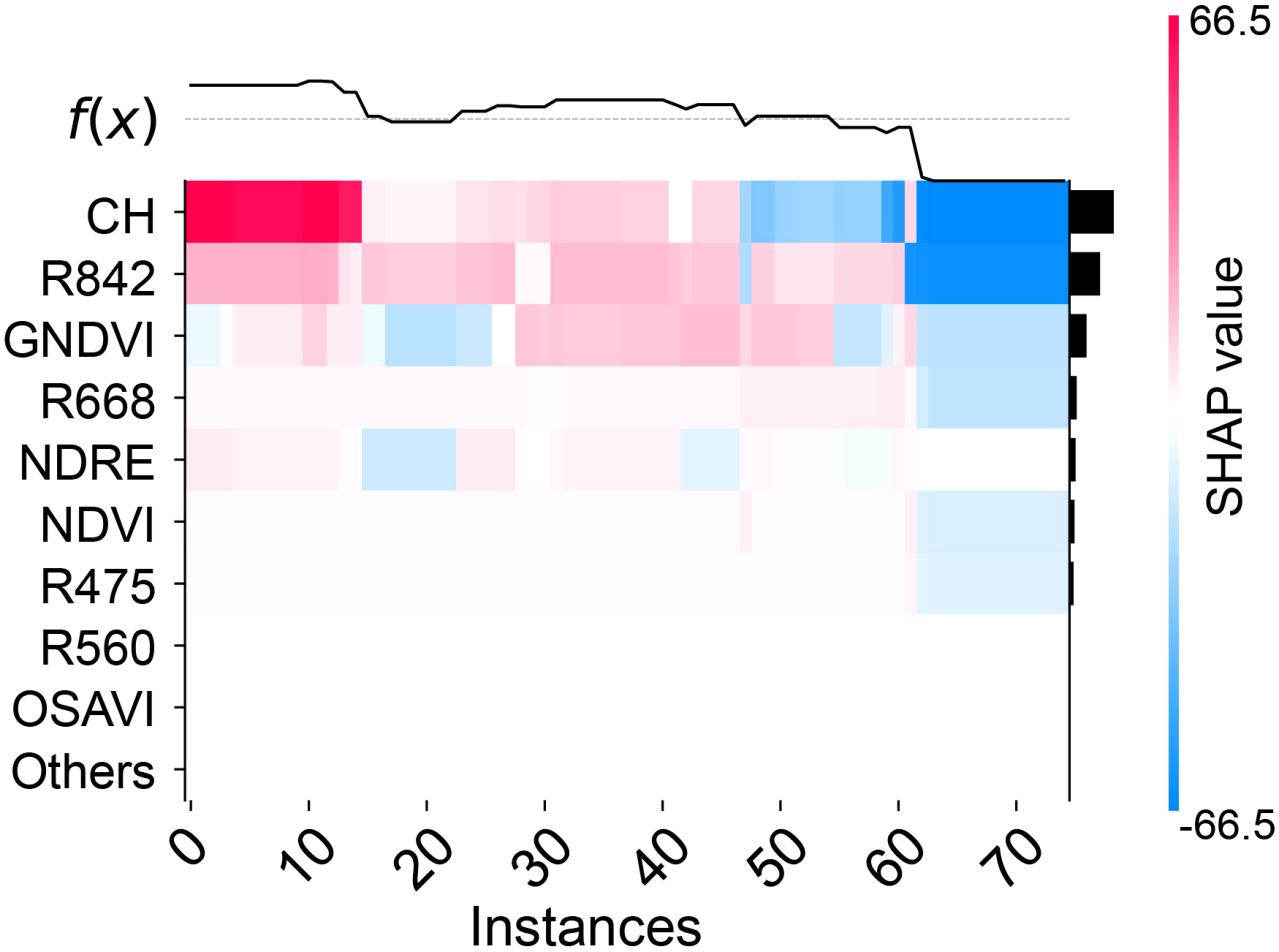
SHAP heatmap plot for the top nine most important variables. The horizontal axis represents arbitrary, unordered test samples. The variations in SHAP values across the horizontal axis reflect the model’s adaptive feature selection and dynamic response across different sample-specific biomass conditions. The color bar represents the SHAP value, with red indicating a positive contribution and blue indicating a negative contribution to the model output. The horizontal axis represents arbitrary test samples. For abbreviations of the variables, refer to the caption of **Figure 5**.

We further present the SHAP heatmap of the top nine important variables in DBAFN, as shown in **Figure 6**. The contribution of each feature varies clearly across samples, indicating strong sample-dependent effects. CH shows the strongest contribution in most instances. In the samples on the left side of the heatmap, CH has large positive SHAP values, suggesting that it strongly promotes biomass prediction in these cases. However, in the samples on the right side, its contribution becomes negative, indicating that the effect of canopy height is not constant across the full sample range.

R842 and GNDVI also show strong contributions in many instances. Their SHAP values are mainly positive in the middle part of the sample sequence, but negative effects appear in several samples, especially in the later part. This pattern suggests that these variables play an important role in distinguishing differences among biomass levels. Compared with CH, R668, NDRE, NDVI, R475, and R560 have relatively weaker contributions, but they still affect the prediction in specific samples. OSAVI and the remaining variables show limited influence overall.

The change in the model output curve at the top of the heatmap is generally consistent with the combined effects of the main variables. Positive SHAP values of CH, R842, and GNDVI are associated with higher model outputs, while negative contributions from these variables are related to lower outputs. Overall, the heatmap indicates that DBAFN does not rely on a single feature. Instead, it adjusts the contribution of key structural and spectral variables across samples, which helps improve the flexibility and robustness of leaf biomass estimation.

### 3.3 Ablation study

**Figure 7:**
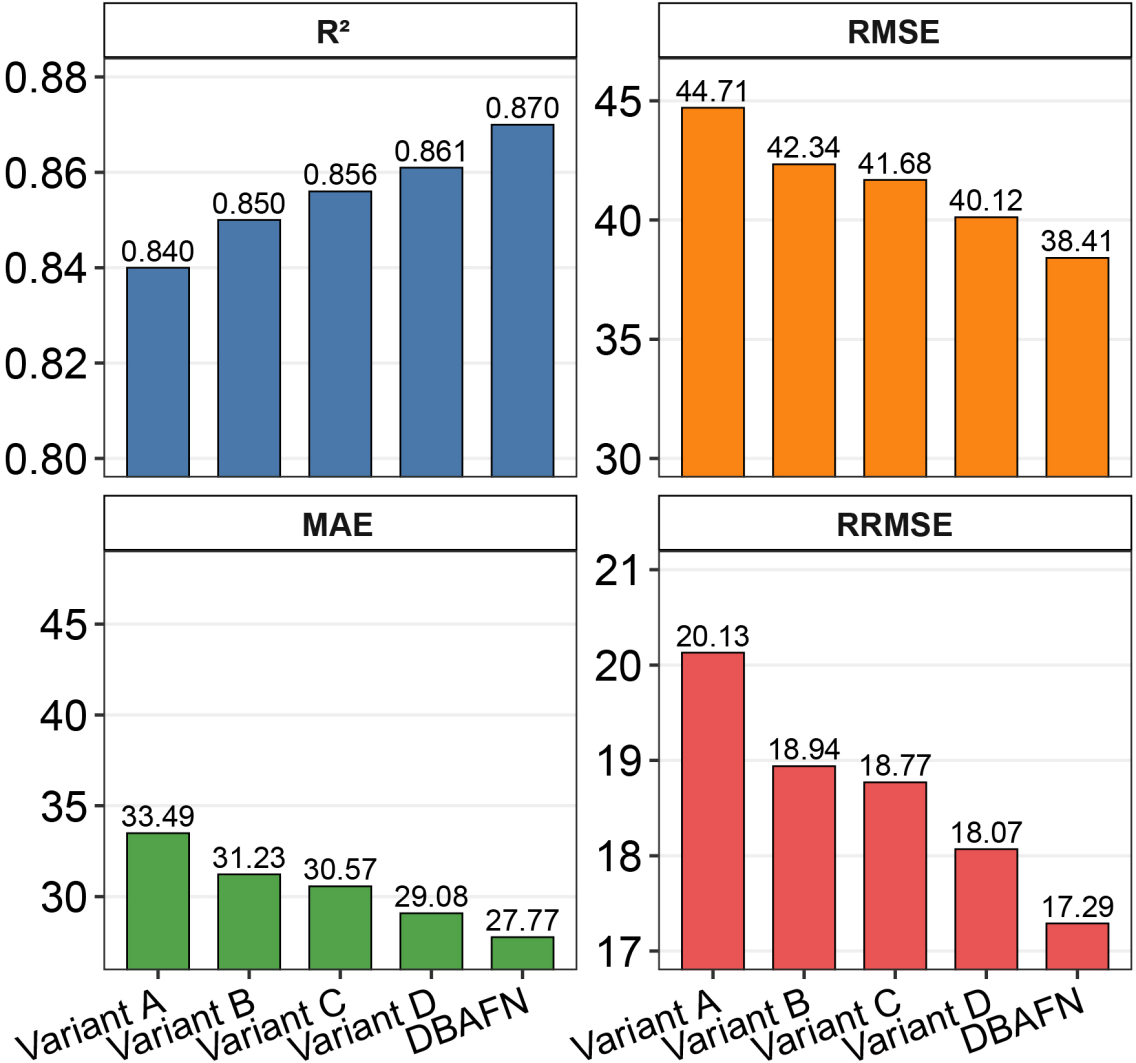
Prediction performance of the ablation experiments. Variant A denotes the model without the dual-branch module; Variant B denotes the model without the hybrid attention module; Variant C denotes the model without the Transformer encoder; Variant D denotes the model without cross-layer feature fusion. See the caption of **Figure 4** for definitions of R², RMSE, MAE, and RRMSE.

An ablation study was conducted to evaluate the contribution of each module in the proposed DBAFN (**Figure 7**). When the dual-branch temporal module was replaced by a single-branch structure (Variant A), the performance decreased significantly. This Variant A model obtained an R^2^ of 0.84, with RMSE, MAE, and RRMSE increasing to 44.71 g/m^2^, 33.49 g/m^2^, and 20.13%. This indicates that multi-scale temporal modeling is essential for capturing both short-term fluctuations and long-term trends. Removing the hybrid attention module also led to a clear performance drop (Variant B), which achieved an R^2^ of 0.85, with RMSE of 42.34 g/m^2^, MAE of 31.23 g/m^2^, and RRMSE of 18.94%. This result suggests that adaptive feature selection helps improve prediction accuracy and robustness. When the Transformer encoder was replaced by fully connected layers (Variant C), which showed a moderate decrease in performance, with an R^2^ of 0.856, RMSE of 41.68 g/m^2^, MAE of 30.57 g/m^2^, and RRMSE of 18.77%. This confirms the importance of modeling long-range temporal dependencies. Without cross-layer feature fusion, Variant D achieved an R^2^ of 0.861, with RMSE, MAE, RRMSE of 40.12 g/m^2^, 29.08 g/m^2^, and 18.07, respectively. The slight decline indicates that integrating multi-level temporal features contributes to more stable predictions. Overall, all variants performed worse than the full model. The dual-branch module and hybrid attention provided the largest improvements, while the Transformer and cross-layer fusion further enhanced model performance.

### 3.4 Independent validation

**Figure 8:**
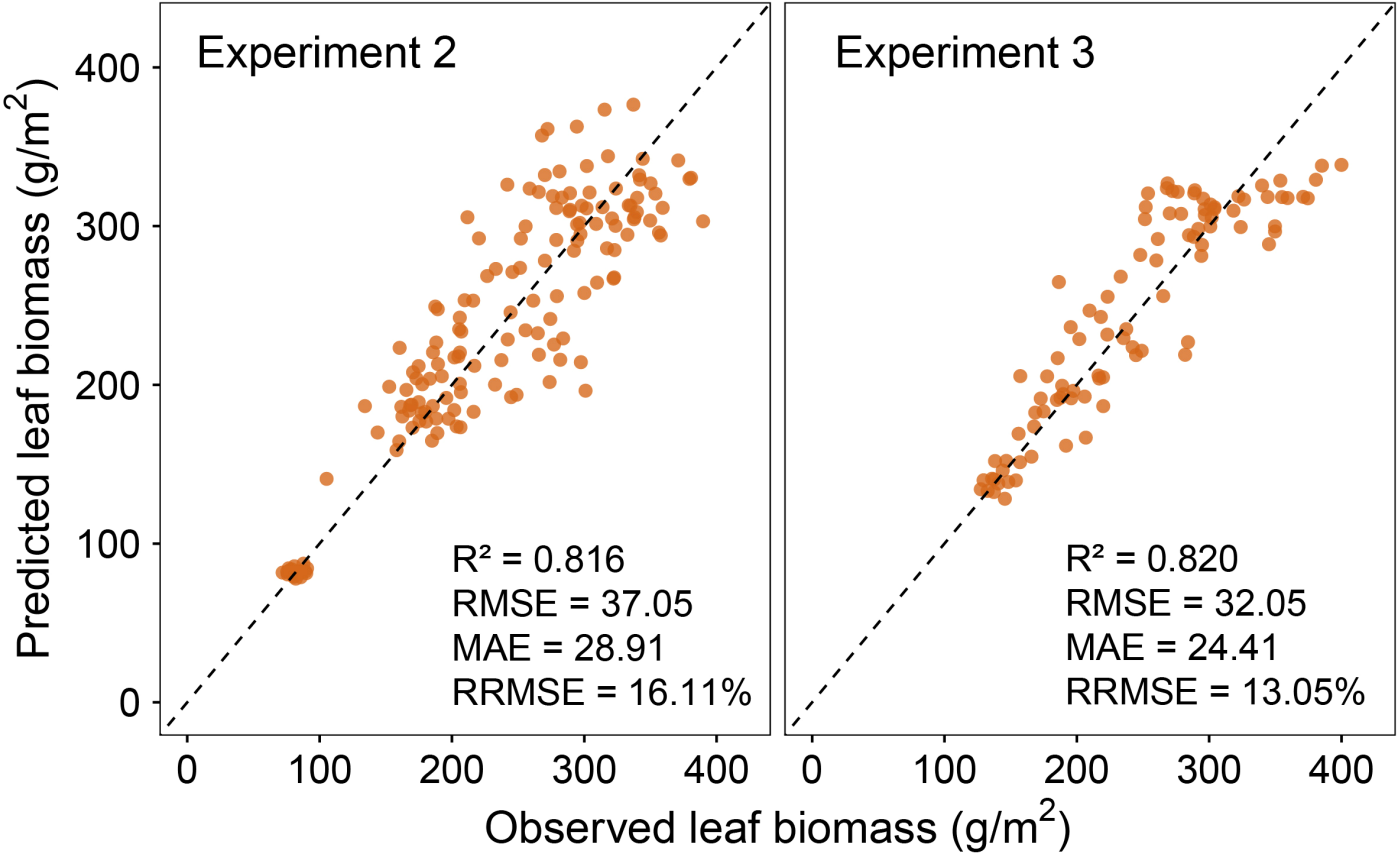
Independent validation results of wheat leaf biomass prediction.

To further evaluate the generalization capability and robustness of the proposed DBAFN model, independent validation was conducted using data from Experiment 2 and Experiment 3 (**Figure 8**), which were collected from a different ecological region (Hebi) under natural growth conditions without nitrogen treatment interventions. **Figure 8** presents the scatter plots of observed versus predicted wheat leaf biomass for both experiments. For Experiment 2, which included 20 diverse wheat genotypes across 40 plots, the model achieved an R² of 0.816, an RMSE of 37.05 g/m², an MAE of 28.91 g/m², and an RRMSE of 16.11%. For Experiment 3, consisting of 25 plots of a single cultivar, the model yielded improved performance with an R² of 0.820, an RMSE of 32.05 g/m², an MAE of 24.41 g/m², and an RRMSE of 13.05%. These results demonstrate that the DBAFN model maintains satisfactory prediction accuracy across different genotypes, ecological regions, and experimental designs, with slightly better performance under homogeneous genetic background (Experiment 3) compared to diverse genotypic conditions (Experiment 2). The consistent agreement between predicted and observed values across both independent datasets confirms the strong generalizability and transferability of the proposed framework for wheat leaf biomass estimation.

## 4 Discussion

### 4.1 Effects of multi-source feature integration

The results in **Figure 3** show that combining R, VI, and CH leads to the highest prediction accuracy, whereas using single-source variables results in significant performance decrease. Among the input variables, CH consistently provides the largest contribution, which is further supported by the SHAP analysis (**Figure 5**). This indicates that structural information plays a dominant role in wheat leaf biomass estimation.

From an agronomic perspective, canopy height directly reflects vertical canopy development and biomass accumulation, while spectral features capture physiological properties such as chlorophyll content and nitrogen status (Li et al., 2021). The complementary nature of these variables explains the observed improvement when they are used together. In particular, spectral indices such as GNDVI and NDVI are sensitive to vegetation vigor but tend to saturate at high biomass levels, whereas CH maintains sensitivity across growth stages (Vera-Esmeraldas et al., 2025; Tian et al., 2025). The SHAP results further confirm that CH, R842, and GNDVI jointly dominate model predictions, suggesting that both structural and physiological information are essential.

Overall, these findings indicate that wheat biomass is jointly controlled by canopy structure and physiological processes. Therefore, integrating multi-source features provides a more comprehensive representation of crop growth, which is critical for improving estimation accuracy.

### 4.2 Importance of temporal modeling in biomass estimation

The ablation results (**Figure 7**) highlight the critical role of temporal modeling in the proposed framework. Removing the dual-branch temporal module leads to the largest performance degradation, indicating that multi-scale temporal dynamics are essential for accurate biomass estimation. In contrast, replacing the Transformer encoder results in a moderate performance decline, suggesting that long-range temporal dependency modeling provides additional but complementary benefits.

Wheat growth is inherently a dynamic process characterized by both short-term fluctuations and long-term phenological progression. Short-term variations are often driven by environmental factors such as irrigation, fertilization, and temperature changes, while long-term trends reflect the transition between growth stages, such as tillering, stem elongation, and grain filling (Aurbacher et al., 2013; Bernacchi et al., 2023; Si et al., 2023). Traditional models typically treat temporal information as independent snapshots or aggregated features, which limits their ability to capture such dynamics.

The dual-branch temporal module addresses this issue by explicitly modeling both short-term and long-term patterns through different receptive fields. This design enables the model to capture fine-scale vegetation changes as well as broader phenological trends. In addition, the Transformer encoder further enhances temporal representation by modeling the dependencies and cumulative effects across the discrete phenological stages within the growing season. This is particularly important because early-season conditions can have cumulative effects on later biomass accumulation (Salvagiotti and Miralles, 2008). These results suggest that temporal dynamics should not be treated as auxiliary information but rather as a primary driver of biomass estimation. Explicit modeling of temporal processes is therefore essential for accurately representing crop growth.

### 4.3 Role of hybrid attention and model interpretability

The HA mechanism also plays an important role in improving model performance. As shown in the ablation study, removing the HA module results in a clear decrease in prediction accuracy, indicating that adaptive feature selection is critical for biomass estimation. Unlike conventional approaches that treat all input features equally, the HA module dynamically adjusts channel importance based on temporal patterns. The combination of average pooling and max pooling allows the model to capture both gradual growth trends and critical events, such as rapid leaf expansion or stress responses. This design aligns with the biological characteristics of crop growth, where both continuous development and abrupt changes influence biomass accumulation (Dreccer et al., 2013; Singh et al., 2024).

The SHAP analysis further supports the effectiveness of this mechanism. The varying contributions of features across samples (**Figure 6**) indicate that the importance of input variables is not constant but depends on growth stage and biomass level. This suggests that the model implicitly performs stage-aware feature selection, which is consistent with agronomic knowledge that different variables dominate at different phenological stages (Zhao et al., 2022). Therefore, the HA module not only improves prediction accuracy but also enhances model interpretability by aligning feature importance with underlying crop growth processes. This ability to learn agronomically meaningful patterns without explicit supervision represents a key advantage of the proposed framework.

### 4.4 Implications, limitations, and future work

The proposed framework has important implications for UAV-based crop monitoring and precision agriculture. By effectively integrating multi-source features and temporal dynamics, the model provides more accurate and stable biomass estimates, which can support applications such as nitrogen management, yield prediction, and crop growth monitoring. In addition, the results demonstrate the value of UAV observations across multiple key growth stages, as the temporal coverage at critical phenological phases enables characterization of crop growth processes.

Despite these advantages, several limitations should be acknowledged. First, while the independent validation across two ecological regions (Jiaozuo and Hebi) demonstrates promising generalizability, the study is still limited within Henan Province. Crop growth patterns can vary significantly across years due to inter-annual climate variability (e.g., drought, heat stress). Further validation using multi-year datasets from a broader range of agro-ecological zones is therefore necessary to fully establish the model’s robustness across diverse temporal and spatial scales. Crop growth patterns can vary across environments, and further validation is needed under different climatic and management conditions (Peng et al., 2020). Second, only spectral and structural features derived from UAV data were considered, while other important factors such as soil properties and meteorological conditions were not included. Incorporating multi-source data could further improve model performance. Third, the proposed framework involves relatively high computational complexity compared to traditional models, which may limit its applicability in real-time scenarios.

Future research should focus on improving model generalization across regions and seasons, as well as integrating additional data sources such as climate and soil information. Furthermore, developing lightweight versions of the model could facilitate real-time deployment in operational agricultural systems. Exploring the transferability of the proposed temporal modeling framework to other crops and agronomic traits also represents a promising direction.

## 5 Conclusions

This study developed a DBAFN framework for estimating wheat leaf biomass using UAV time-series data. The proposed model was designed to address key challenges in biomass estimation, including multi-scale temporal dynamics, adaptive feature selection, and long-range dependency modeling. The results demonstrate that DBAFN achieves superior performance compared with conventional machine learning models. The model obtained an R² of 0.87, RMSE of 38.41 g/m², MAE of 27.77 g/m², and RRMSE of 17.29%, indicating high accuracy and stability. In addition, multi-source feature analysis shows that the integration of reflectance, vegetation indices, and canopy height provides the most comprehensive representation of wheat growth, with canopy height playing a dominant role. SHAP-based interpretation further confirms that structural and key spectral features jointly contribute to biomass estimation. The ablation experiments reveal the contribution of each model component. The dual-branch temporal module significantly improves the ability to capture multi-scale temporal variations, while the hybrid attention mechanism enhances adaptive feature selection by emphasizing informative signals and suppressing noise. The Transformer encoder and cross-layer feature fusion further improve the modeling of long-range temporal dependencies and enhance prediction stability. These findings indicate that explicitly modeling temporal dynamics is essential for accurately representing crop growth processes. The proposed framework provides important implications for UAV-based crop monitoring and precision agriculture, particularly in biomass estimation and nitrogen management. Furthermore, independent validation across different ecological regions and varied genotypic conditions confirms the strong generalization capability of DBAFN, with R² values of 0.816 and 0.820 for Experiment 2 and Experiment 3, respectively, demonstrating its potential for broader application in precision agriculture. Future research should focus on improving model generalization across different environments, integrating additional data sources such as climate and soil information, and developing lightweight models for real-time applications. In generally, this study demonstrates that integrating multi-source information with temporal modeling provides an effective approach for improving wheat leaf biomass estimation.

## Data Availability

The original contributions presented in the study are included in the article/supplementary material. Further inquiries can be directed to the corresponding author.
